# Unique core genomes of the bacterial family *vibrionaceae*: insights into niche adaptation and speciation

**DOI:** 10.1186/1471-2164-13-179

**Published:** 2012-05-10

**Authors:** Tim Kahlke, Alexander Goesmann, Erik Hjerde, Nils Peder Willassen, Peik Haugen

**Affiliations:** 1 Department of Chemistry, Faculty of Science and Technology, The Norwegian Structural Biology Centre, University of Tromsø, 9037, Tromsø, Norway; 2 Center for Biotechnology (CeBiTec), Institute for Bioinformatics, Bielefeld University, Bielefeld, Germany

## Abstract

**Background:**

The criteria for defining bacterial species and even the concept of bacterial species itself are under debate, and the discussion is apparently intensifying as more genome sequence data is becoming available. However, it is still unclear how the new advances in genomics should be used most efficiently to address this question. In this study we identify genes that are common to any group of genomes in our dataset, to determine whether genes specific to a particular taxon exist and to investigate their potential role in adaptation of bacteria to their specific niche. These genes were named *unique core genes*. Additionally, we investigate the existence and importance of unique core genes that are found in isolates of phylogenetically non-coherent groups. These groups of isolates, that share a genetic feature without sharing a closest common ancestor, are termed *genophyletic* groups.

**Results:**

The bacterial family *Vibrionaceae* was used as the model, and we compiled and compared genome sequences of 64 different isolates. Using the software orthoMCL we determined clusters of homologous genes among the investigated genome sequences. We used multilocus sequence analysis to build a host phylogeny and mapped the numbers of unique core genes of all distinct groups of isolates onto the tree. The results show that unique core genes are more likely to be found in monophyletic groups of isolates. Genophyletic groups of isolates, in contrast, are less common especially for large groups of isolate. The subsequent annotation of unique core genes that are present in genophyletic groups indicate a high degree of horizontally transferred genes. Finally, the annotation of the unique core genes of *Vibrio cholerae* revealed genes involved in aerotaxis and biosynthesis of the iron-chelator vibriobactin.

**Conclusion:**

The presented work indicates that genes specific for any taxon inside the bacterial family *Vibrionaceae* exist. These unique core genes encode conserved metabolic functions that can shed light on the adaptation of a species to its ecological niche. Additionally, our study suggests that unique core genes can be used to aid classification of bacteria and contribute to a bacterial species definition on a genomic level. Furthermore, these genes may be of importance in clinical diagnostics and drug development.

## Background

The separation of bacteria into discrete taxa is still a matter of controversy in biological systematics. Notably a universal definition of bacterial species, as it exists for eukaryotes, is an issue of ongoing debate. The ability of bacteria to acquire genes horizontally, as well as the ability to lose vast numbers of genes when adapting to a specific niche, raises the question if such a definition even exists [1-3].

**Figure 1:**
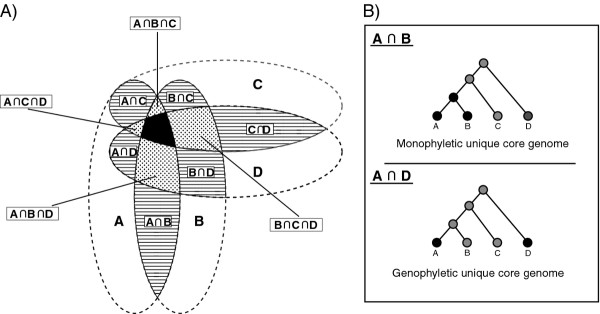
**Unique core genomes of a group of isolates. (A)** Shown is the pan-genome of four genomes A, B, C and D (dashed lines) from different taxa with its core-genome (black area), unique genes of each genome (white areas) and the accessory genome (hatched and dotted areas). The accessory genome is composed of unique core genomes, i.e. the intersections of the gene content of all combinations of sub-groups of genomes. The number of possible combinations for n genomes is $2^{n}-1$ (including the core-genome and the set of unique genes of each genome). Hence, the maximum number of genome combinations of sub-groups that can form a unique core genome is $2^{n}-\left(n+1\right)$. In case of n=4 a maximum of 10 different unique core genomes can be formed as the intersection of either two (hatched areas) or three different genomes (dotted areas). The size of each unique core genome is given by the number of homolog clusters shared by the particular group of isolates. **(B)** Unique core genes are found either in isolates of phylogenetically coherent, i.e., monophyletic groups or in groups of isolates that did not derive from a closest common ancestor (genophyletic).

One concept from the early years of genomics is the *differential genome comparison* where genomes are compared as "bags of genes" [4] to identify differences in the gene content of related isolates. It was hypothesized that the genes found in only one species or isolate might play an important role in the development of a specific phenotype [5]. However, one problem in the beginning of the genomic era was a clear prevalence of sequencing projects that focussed on bacterial pathogens. This limited the possibility to determine genetic features that are present in all representatives of one taxon, given that only a small fraction of the bacterial diversity is represented by pathogenic strains. But new time- and cost efficient sequencing technologies made it possible to sequence high numbers of non-pathogenic isolates, covering the entire spectrum of the genetic diversity of a taxon. In 2005 Tettelin *et al.* tried to describe a species by building its so called *pan-genome*[6]. They defined the pan-genome of multiple bacterial genomes as a union of three distinct sets of genes: genes found in all investigated genomes (core genome), genes found in just one isolate (unique genes) and genes found in more than one but not all members of the investigated group (accessory genome). Subsequent pan-genome studies revealed that high numbers of core-genes exist for all investigated taxa, whether species, genus or family [7-11]. Thus, using the pan-genome terminology, shared phenotypical traits should be reflected by genes included in the core-genome of a group of bacteria [12]. Furthermore, picking up the idea of differential genome comparison, phenotypical traits that are specific to a group of bacteria, phylogenetically coherent or not, should be reflected by its *unique* core genome, i.e., core genes that are unique to this group in comparison to other, closely related organisms (Figure 1). It seems legitimate to assume that these unique core genes exist for certain groups of bacteria. But the question remains whether these genetic traits follow a phylogeny, i.e., are found in phylogenetically coherent groups of organisms, or whether they are distributed over isolates of various taxonomic clades without a close common ancestor. Horizontal gene transfer (HGT) as well as the loss of genes may lead to the same pheontype in a phylogenetically diverse group of isolates. In this article, we will term groups of isolates that share a genetic trait or phenotype but have no closest common ancestor *genophyletic* groups in comparison to monophyletic groups, where all isolates are derived from a closest common ancester (Figure 1B). For pathogenic bacteria the *distributed genome hypothesis* states that HGT is a major driving force in evolution of these phenotypes, indicating that unique core genes of pathogens may frequently be found for genophyletic groups [13]. However, a recently published phylogenomics study also states the existence of unique core genes for all investigated monophyletic groups of bacteria [14].

xIn the presented study we compared 64 *Vibrionaceae* genomes to address the question whether unique core genes exists inside this bacterial family and whether they appear more often in monophyletic than in genophyletic groups. Given that unique core genes exist, the cellular processes these genes are involved in, can provide knowledge about niche adaptation and development of specific phenotypes. In case of unique core genomes of monophyletic groups, these genes may also provide a way to rapidly classify bacteria into different species as proposed by Dutilh *et al.*[14] which is of particular interest not only for taxonomists but for the development of clinical and diagnostic tests. Additonally, they are promissing targets for the development of vaccines and antibiotics specific for a discrete group of bacterial organisms.

For our analysis we chose the family *Vibrionaceae* of gamma-proteobacteria because it is a diverse group that currently encloses 130 species from seven genera, including *VibrioAliivibrio* and *Photobacterium*, and they are typically abundant in aquatic environments (i.e., in oceans, in freshwaters and in brackish waters) [15]. Historically, representatives of the *Vibrionaceae* family have attracted considerable attention because of their abilities to cause serious diseases in humans (e.g., *V. choleraeVibrio parahaemolyticus* and *Vibrio vulnificus*), for example after consumption of undercooked seafood or intake of contaminated water. Despite the infamous reputation of *Vibrionaceae*, the majority of these bacteria are normally harmless to healthy humans animals and play important roles in their natural habitats, for example in the regeneration of nutrients.

## Results and discussion

### Genome dataset

Table 1 summarizes the genome dataset used in the presented study (see Additional File 1 for complete list). It comprises 64 genome sequences from the bacterial family *Vibrionaceae*, and includes representatives from 20 species that are distributed into the *Vibrio*, *Aliivibrio* and *Photobacterium* genera (five genomes without a species assigned). In addition to 62 *Vibrionaceae* genomes, that were publicly available when this study was initiated, the genome sequences of *Aliivibrio wodanis* str. 06-09-139 and *Vibrio anguillarum* str. NB10, obtained from our own sequencing projects, were also included. Thirteen of the genome sequences are completely assembled: nine, three and one of which belong to the *Vibrio*, *Aliivibrio* and *Photobacterium* genera, respectively. The dataset includes pathogenic as well as non-pathogenic organisms from 18 clinical and 42 environmental isolates. The origin of the remaining four genomes could not be verified. Genome sequences of 19 different *Vibrionaceae* species are included, of which nine are represented by more than one strain. Of these are six species represented by either just pathogenic or non-pathogenic organisms, whereas the three species *V. cholerae*, *Vibrio alginolyticus* and *Vibrio harveyi* contain both types.

**Table 1:** Dataset composition summary

**Organism**	**# Genomes**				
		**Pathogenic**	**Non-pathogenic**	**Pathogenic**	**Non-pathogenic**
***Aliivibrio***;					
* A. fischeri*	2	-	2	-	-
* A. salmonicida*	1	1	-	-	-
* A. wodanis*	1	1	-	-	-
***Vibrio***;					
* V. alginolyticus*	2	1	1	-	-
* V. anguillarum*	1	1	-	-	-
* V. campbellii*	1	-	1	-	-
* V. cholerae*^1^	26	11	2	11	-
* V. coralliilyticus*	1	1	-	-	-
* V. furnissi*	1	-	-	1	-
* V. harveyi*	3	2	1	-	-
* V. metschnikovii*	1	-	-	-	1
* V. mimicus*	3	2	-	1	-
* V. orientalis*	1	-	-	1	-
* V. parahaemolyticus*^1^	6	2	-	2	-
* V. splendidus*	2	2	-	-	-
* V. shilonii*	1	1	-	-	-
* V. vulnificus*	2	-	-	2	-
* V.* sp.	4	1	3	-	-
***Photobacterium***;					
* P. angustum*	1	-	1	-	-
* P. damselae*	1	-	1	-	-
* P. profundum*	2	-	2	-	-
* P* sp.	1	-	1	-	-

Composition of the dataset used in this study. A complete list of all 64 genomes can be found in Additional File 1.

^1^The origin of two isolates, whether environmental or clinical, could not be determined.

In summary, we compiled a large dataset which includes genome sequences from 64 representatives of the bacterial family *Vibrionaceae*. Pathogenic and non-pathogenic organisms are included as well as environmental and clinical isolates, covering a wide spectrum of the genetic diversity of this family.

### Identification of unique core genomes

The sequences of 63 isolates from our dataset were subjected to the Glimmer gene prediction software [16] to provide gene predictions of equal and high quality. The genome sequence of *Aliivibrio salmonicida* was manually curated in our group [17] and used as a template for annotation later in this study. In total the predictions identified 207,403 protein coding sequences in all 64 isolates.

Clustering of homologous genes was performed using orthoMCL [18] on the translated protein sequences of all predicted genes. As our dataset comprises relatively closely related organisms we chose a conservative parameter value of 50% sequence identity for the clustering. Additionally, to minimize changes in the clustering that are based on software parameters, we performed multiple orthoMCL runs with varying parameter values and excluded those clusters that were not stable among all conditions (see Methods). For the identification of unique core genomes we excluded those homology clusters that either contained all 64 or just duplicates from one isolate. In total, we identified 12,914 clusters of unique core genes in our dataset that are stable over all orthoMCL runs performed. They containing a total number of 201,329 protein sequence, i.e., 74% of all protein sequences in the dataset. The different unique core genomes were then determined by identification of those homolog clusters that contain protein sequences of the exact same isolates. This resulted in 4,557 different combinations of 2 to 63 isolates that shared at least one unique gene. Hence that the number of homology clusters included in each unique core genome also denotes the minimal number of genes per isolate included in it.

**Figure 2:**
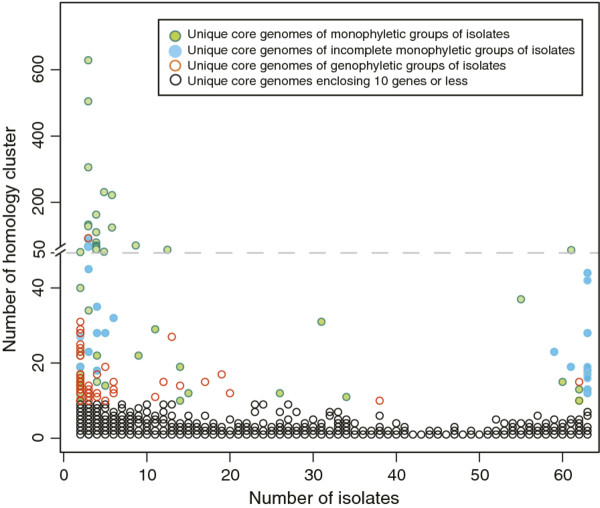
**Distribution of unique core genomes.** The number of homology clusters and isolates included in each determined unique core genome are shown. Given that at least one gene of each isolate has to be included in a homology cluster of a unique core genome, the number of homology clusters denotes the minimum number of different genes per isolate included in a unique core genome. Black circles represent unique core genomes including 10 or less homology clusters of any group of isolates. Unique core genomes including more than 10 homology clusters are colored according to the legend. Hence that for better visibility, the scaling factor changes for unique core genomes enclosing more than 50 homology cluster.

We sub-divided all unique core genomes that contain more than 10 homology clusters into 3 distinct groups: monophyletic groups of isolates, genophyletic groups of isolates and coherent phylogenetic groups with one isolate missing (incomplete monophyletic groups). The third group of incomplete monophyletic groups was introduced to accommodate the fact that the majority of the genome sequences included in this study are not fully assembled and thus might lack genes although they are present in the complete genome sequence. Figure 2 summarizes the distribution of unique core genomes in our dataset based on the number of homology clusters and isolates included. It shows that the vast majority (4,439 or 94%) of the unique core genomes found contains at most 10 homology clusters. Another expected observation is that the amount of homology clusters, and thus the number of genes per isolate, decreases with increasing number of isolates included. Of the 118 unique core genomes that contain 11 homology clusters and more, 39% (46) contain only two isolates. Of these, 24 (52%) are found in genophyletic groups of isolates, showing that unique core genomes of few isolates are found in equal numbers in monophyletic and genophyletic groups. Furthermore, among the 72 unique core genomes that are formed by groups of at least 3 isolates and that contain more than 10 homology clusters, 22 (30%) are found in genophyletic groups of isolates. Thus, an increasing number of isolates per unique core genome decreases the fraction of unique core genomes of genophyletic groups. Another major finding is that unique core genomes of genophyletic groups rarely exceed 35 homology clusters whereas almost 50% of all unique core genomes of monophyletic groups include >50 homology cluster.

### Phylogenetic relationships among the investigated genomes

We wanted to evaluate the clusters of homologous genes, as identified by orthoMCL, in a phylogenetic context, and therefore constructed a robust phylogeny based on the nucleotide sequences of six core genes (*uvrDdefBrsmBpmbAglnA* and *dapA*). The genes were selected based on criteria as recommended by Zeigler (2003) [19]. The sequences of all six genes were concatenated and aligned using MAFFT v. 6.833 [20] to produce a final dataset of 64 sequences of 7,674nt in length. Phylogenetic analyses were carried out using the Epos framework v. 0.9 [21]. Maximum-Likelihood (ML) phylogeny of the sequences was constructed using RaxML v. 7.0.4 [22] and teStamatakis2006 Bayesian inference of phylogeny was done using MrBayes v. 3.1.2 [23,24].

**Figure 3:**
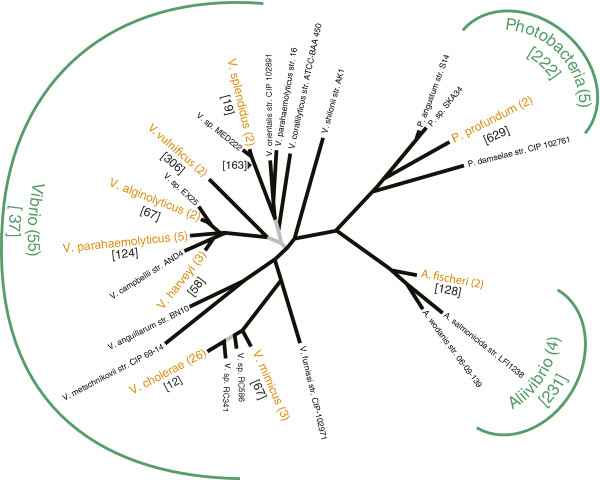
**Unrooted phylogeny of the bacterial family vibrionaceae.** The tree is a summary of a phylogeny based on six core genes (uvrD,defB, rsmB, pmbA, glnA and dapA) from 64 genomes. The number of representatives of each species and genera are shown in parentheses. Branches that are highly supported by statistical analyses (i.e., ≥90% bootstrap support and ≥ Bayesian posterior probability (PP)) are shown in black, whereas grey branches are moderately supported (i.e., 80-90% Bootstrap support or 0.8-0.9 PP). The numbers of unique core genes are shown in brackets.

Figure 3 shows the resulting ML-tree, which is in agreement with the best MrBayes tree (the Robinson-Foulds distance [25] between the teRobinson1981 ML-tree and the best Bayesian tree was calculated to 0.18). The overall topology is highly supported by ML-bootstrap and Bayesian analyses, and, except for *V. parahaemolyticus* and *Vibrio splendidus*, the evolutionary relationships between species and genera are well resolved. Five *V. parahaemolyticus* strains are found clustered on the same major branch with *V. harveyiVibrio campbelliiV. alginolyticus* and t*Vibrio* sp. EX25 as closest neighbors, whereas *V. parahaemolyticus* str. 16 is sister to *Vibrio orientalis* str. CIP 102891. The well supported placement of *V. parahaemolyticus* str. 16 separate from the other strains strongly suggests that the latter should be renamed. The same phylogenetic relationship was recently noted by Vesth *et al.*[9]. Moreover, our analysis together with previous analyses [26,27] support that *V. sp.* MED222 is closely related to *V. splendidus*, and perhaps should be named accordingly.

After reconstruction of the *Vibrionaceae* tree (Figure 3) the size of the unique core genomes of three genera (*Vibrio*, *Photobacteria* and *Aliivibrio*) and nine species was mapped onto the phylogeny. The size of an unique core genome represents the number of homology clusters that are unique to a specific group of isolates. Each genus includes fifty-five (*Vibrio*), five (*Photobacterium*) and four (*Aliivibrio*) genomes. For species, the corresponding numbers were two (*A. fischeri*, *P. profundum*, *V. alginolyticus*, *V. alginolyticus* and *V. splendidus*), three (*V. harveyi* and *V. mimicus*), five (*V. parahaemolyticus*) and twenty-six (*V. cholerae*). For all investigated phylogenetic lineages we found sets of core genes not found in any isolate outside the clade. For the three genera *Vibrio*, *Photobacterium* and *Aliivibrio* the synapomorphic unique core genomes consist of 37, 222 and 231 genes, respectively. The corresponding numbers for the nine species are 12 unique core genes for V. cholerae, 67 for *V. mimicus*, 58 for *V. harveyi*, 124 for *V. parahaemolyticus*, 67 for *V. alginolyticus*, 306 for *V. vulnificus*, 19 for *V. splendidus*, 629 for *P. profundum* and 128 uniquecore genes for *A. fischeri*. Additionally, we calculated the size of the unique core genome of both *V. splendidus* isolates and strain *V. sp.* MED222, which included 163 unique core genes.

In summary, our results show that unique core genomes exist for all investigated taxa of the bacterial family *Vibrionaceae*. Although this was already recently suggested for any bacterial taxa we could show that unique core genes can be identified even when comparing high numbers of closely related isolates of a single bacterial family [14].

### Tracking of unique core genes on the vibrionaceae phylogeny reveals local maxima at taxon borders

**Figure 4:**
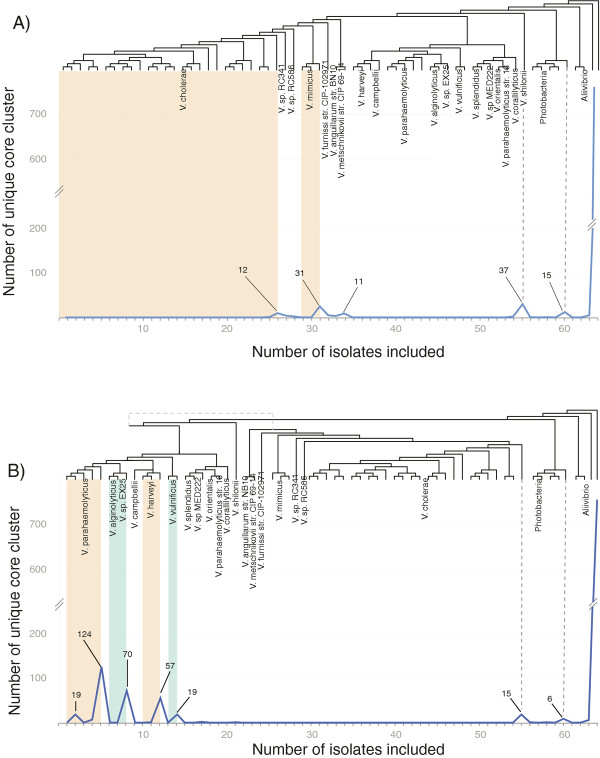
**Correlation of the unique core genome size and phylogeny.** The number of unique core genes was calculated when starting from different end nodes and then successively adding more genomes according to the phylogenetic tree (see Figure 3). Depending on the starting point for the calculations, local maxima are typically found when all genomes of a taxon (or all taxa with the same phylogenetic distance to the starting point) are added. When starting from **(A)** V. cholerae or **(B)** V. parahaemolyticus strains of most recent origins, then the first local maxima were found after inclusion of all strains of the respective Vibrio species (i.e., 12 and 124 genes, respectively). With *V. cholerae* as the starting point succeeding maxima were found after inclusion of all representatives of *V. mimicus* and two additional representatives of the *Vibrio* species(31), the genus *Vibrio* (37) and the genus *Photobacterium* (15). When starting with *V. parahaemolyticus* the corresponding local maxima were found after inclusion of all strains of *V. parahaemolyticus* (124), *V. alginolyticus* and V. sp. EX25 (70), *V. harveyi* (57) and *V. vulnificus* (19), the complete genus *Vibrios* (15) and all isolates from genus *Photobacterium* (6).

To investigate in further detail how unique core genomes are distributed on the phylogeny, we calculated the changes in size of a unique core genomes, when starting at one leaf in the phylogenetic tree and successively adding the closest neighbors to our calculation. The numbers of unique core genes were then subsequently mapped onto the *Vibrionaceae* phylogeny. Figure 4 shows two examples where counting of unique core genes started at *V. cholerae* (Figure 4A) or *V. parahaemolyticus* (Figure 4B) strains of most recent origins. Interestingly, the number of unique core genes was close to zero, for the species at which the counting started unless all isolates were included in the dataset. Once all genomes were included a local maximum was typically calculated. For example, when starting from the *V. cholerae* isolate of most recent origin, we observe the first local maximum (i.e., 12 genes) when all *V. cholerae* genomes are included in the calculations (Figure 4A). Furthermore, the next local maximum of 31 unique core genes is found when all the closest neighbors (i.e., *V. mimicus* and the two related isolates *V. sp.* RC341 and *V. sp.* RC586) were added. The next major local maxima are found after inclusion of all representatives of (i) the *Vibrio* genus and (ii) the *Photobacterium* genus. Similarly, we calculated local maxima at taxon borders when starting at the *V. parahaemolyticus* clade (Figure 4B). These figures show that, following the phylogeny, unique core genes are found almost exclusively in monophyletic groups of isolates.

### Unique core genomes of groups of genophyletic isolates

The existence of unique core genomes of isolates that do not share a closest common ancestor can be explained either (i) by loss of genetic features from the majority of representatives of a bigger phylogenetic group or (ii) by HGT. Although HGT can generally be considered more parsimonious than many separate gene deletion events, we wanted to estimate its frequency in unique core genomes of genophyletic groups. We investigated the annotated functions of the unique core genes of one, preferably fully assembled, template isolate per unique core genome and searched for genes with plasmid or phage related functions. Additionally, genes related to pathogenicity were also assumed to indicate HGT as recent studies have shown that HGT plays a major role in the evolution of pathogenic bacteria [1,2,13]. We also investigated the distribution of the unique core genes on the chromosomes of the chosen isolate. We assume that gene loss results in gene artefacts with little or no spatial correlation rather than in clusters of genes found in the same genomic loci. By contrast, horizontally acquired genes are more likely to be found in one genomic loci that has been transferred into the host cell.

In our analysis we identified 46 different unique core genomes that are formed by genophyletic groups containing at least 11 homology clusters. The number of isolates in these groups vary from 2 to 62 with 48% including >2 isolates. Additional File 2 summarizes the functional annotations and numbers of genomic loci the unique core genes of the chosen template isolates are found in. Of all 46 unique core genomes 27 (58%) are found in less than 5 genomic loci on the template. Additionally, unique core genes of genophyletic groups of few isolates tend to be widely distributed in the template sequence. On the other hand, unique core genes of genophyletic groups of >4 isolates are almost exclusively found in single loci on one of the chromosomes. Functional analysis revealed that almost 50% (22) of the genophyletic unique core genomes contain hypothetical proteins or proteins of various ambiguous or unrelated functions. We were able to annotate functions to 17 of the unique core genomes that are found in few genomic loci: seven are mostly associated with plasmid or phage related functions and one encloses all but two genes of the toxin co-regulated pilus gene cluster of *V. cholerae*[28]. Another four unique core genomes of genophyletic groups mostly contain proteins that are associated with secretion systems III, IV or VI and three additional unique core genomes enclose mostly homologs of the flagellar apparatus. Annotation of the remaining unique core genomes revealed genetic loci related to bacterial flagellar or fimbria, purine metabolism and various other functions (see Additional file 3 for more details).

In summary, we found indications that HGT is the origin of most unique core genomes of genophyletic groups, notably when the number of included isolates reaches four. Additionally, our results support previous findings about the impact of HGT on the evolution of pathogenic bacteria, by showing that among the biggest genophyletic groups are those unique core genomes that comprise mostly genes associated with pathogenicity.

### Unique core genes and niche adaptation

In an attempt to link unique core genes of monophyletic groups to behavioral and metabolic traits we further investigated the unique core genome of *V. cholerae*. This species was chosen as a case study because, given that it is the biggest group in our dataset and given the number of closely related isolates it was differentially compared to, we assume that the unique core genes of *V. cholerae* are most likely to stay unique for this species, even if more *Vibrionaceae* genomes are added.

The unique core genome of *V. cholerae* was calculated to comprise 12 genes. Unfortunately the biological role of 8 genes remains unknown or show only poor hits to known functional classes. However, the annotations of the remaining three genes provide more insight into the role of unique core genes for the development of particular phenotypes. One unique core gene is annotated as the aerotaxis protein Aer2 and is part of the class of methyl-accepting chemotaxis proteins. These proteins sense one or several biochemical stimuli and enable motile bacteria to rapidly change their tactic behavior to either move towards the stimulus or away from it [29,30]. Aer2 was recently reported to cause aerotaxic behavior in *V. cholerae*[31]. Aerotaxis, or energy taxis, is the movement of bacteria towards or away from oxygen, a crucial electron acceptor in the energy metabolism of many organisms. This and related energy-tactic behaviors have only been reported for a small number of bacterial species and are discussed as having a major impact on the adaptation of a species to its ecological niche [32]. The ability to navigate towards higher oxygen concentrations may represent a major advantage for *V. cholerae* that populates almost all aquatic environments, including brackish waters.

**Figure 5:**
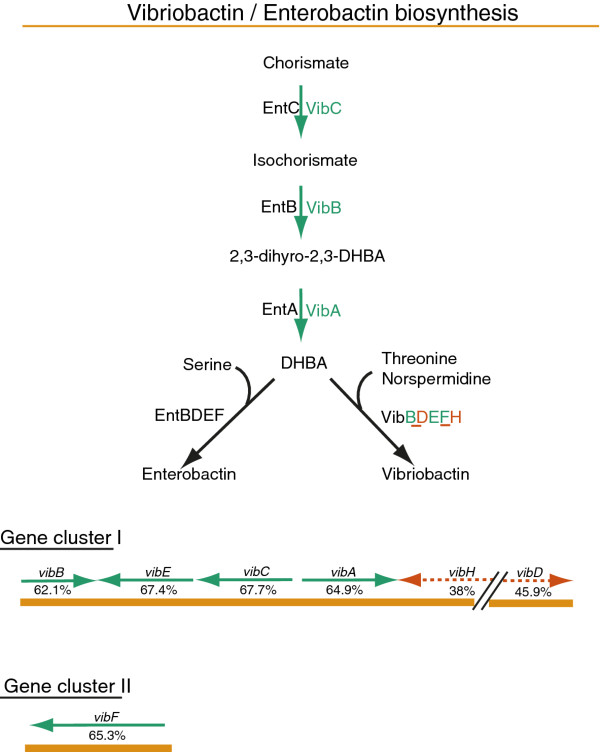
**Vibriobactin/Enterobactin biosynthesis pathway.** The two gene clusters of the vibriobactin biosynthesis are shown. Genes that are part of the unique core genome of V. cholerae are denoted by red dashed arrows. Green arrows indicate genes also found in other isolates of the dataset. Numbers show the percent identity of the best blast hit to a non-V. cholerae isolate in our dataset. The schematic biosynthesis pathways of both siderophores enterobactin and vibriobactin are based on publications by Wyckoff *et al*. [34,36]

The remaining two unique core genes, *vibH* and *vibD* are part of the biosynthesis pathway of the catechol siderophore vibriobactin, which has previously been identified as unique to *V. cholerae*[33,34]. The acquisition of iron is crucial for all aquatic organisms and the ability of utilizing iron through multiple systems was discussed to be important during growth of environmental *V. cholerae* isolates. The biosynthesis of specific iron-chelators in addition to other unspecific siderophores may represent an advantage for the adaptation to a specific niche [35]. Figure 5 shows the biosynthesis pathway of vibriobactin in comparison to the biosynthesis of the closely related enterobactin as proposed by Wyckoff *et al.*[36]. Enterobactin is also a catechol siderophore and is found in several Gram-negative bacteria including *Escherichia coli*[37]. Interestingly, the biosynthesis pathways of the vibriobactin and enterobactin precursor, named DHBA, are identical, and it is the final steps that decide the fate of DHBA to either vibriobactin or enterobactin. The final steps of synthesis of vibriobactin are dependent on the products of *vibH* and *vibD*[36,38]. In other words, the biosynthesis of vibriobactin and enterobactin is dependent on the same set of genes, except for *vibH* and *vibD* that are required for productions of vibriobactin only and are part of the unique core genome of *V. cholerae*.

Together, the genetic traits represented by the unique core genes, that are of known function, may allow optimal acquisition of essential nutritions and elements in the ecological niche of this species, especially in brackish or sewage-contaminated aquatic environments.

## Conclusions

It is widely accepted that adaptation to a specific niche affects genome structure and gene content. The genomic changes may occur through rearrangement of genes and regulatory elements, changes in transcriptional regulation or by HGT and loss of genes. It was recently shown that gene loss and HGT play an important role in the genomes of highly specialized bacteria when adapting to the metabolism of a new host [1,39]. Our results support these findings and additionally indicate that HGT is the main reason for genetic features that are shared among isolates that do not share a closest common ancestor. Nevertheless, our findings show that unique genetic traits are more likely to be shared among monophyletic than among genophyletic groups of isolates. Thus, even if bacterial diversity can mostly be described as ä continous spectrum of genotypic variation" [40] we hypothesize that functional sub-systems exist that discretize this spectrum to an extent, where taxonomical demarcations are possible. This was also recently suggested for other bacterial taxa [14]. Therefore, one cornerstone in a genome-based species definition will be the identification of common and unique functional elements present in monophyletic groups of organisms, with respect to their close relatives. Furthermore, our results revealed that the genes identified to be part of the unique core genome of *V. cholerae* are likely to play an important role in adaptation of this species to its specific ecological niche. Future studies may also investigate the importance of unique core genes of higher taxa, e.g. genera or other monophyletic groups, to examine their role in bacterial taxonomy and evolution. One crucial step in these studies will be the choice of appropriate parameters for homology clustering and determination of uniqueness of genomic traits. This was also discussed for pan-genome analysis’ where the chosen percent identity cut-off can greatly influence the outcome of a study [41].

## Methods

### Genome dataset

XWhen this study was initiated, 62 fully sequenced bacterial genomes were publicly available in the database hosted by the *National Center of Biotechnology Information*. All 62 genome sequences were included in this analysis as well as the available plasmid sequences of six strains: *A. salmonicida* str. LFI1238, *A. fischeri* str. ES114, *A. fischeri* str. MJ11, *P. profundum* str. S99, *V. harveyi* str. ATCC BAA-1116 and *V. vulnificus* str. YJ016. Furthermore two yet unpublished *Vibrionaceae* genomes were included: *A. wodanis* str. 06-09-139 and *V. anguillarum* str. NB10. Both genomes were obtained from ongoing sequencing projects that are carried out in our laboratory in collaboration with other institutions (Dr. Nicholas Thompson and co-workers at Wellcome Trust Sanger Institute, and Prof. Debra Milton and co-workers at University of Umeå, respectively). A complete list of all genome sequence used in the analysis can be found in Additional File 1.

### Gene prediction and annotation

The genomic sequences, either contigs or finished chromosomes and plasmids, of all selected genomes were concatenated to one pseudochromosome per genome. The sequence parts were separated by the spacer sequence 5’-CTAGCTAGCTAG-3’ that contains stop codons in all six reading frames. Genes were predicted using the gene prediction software Glimmer v.3.02 on all but the *A. salmonicida* pseudomolecule. All together our dataset enclosed a total of 64 genomes and 207,403 protein coding sequences.

Annotation of genes was performed subsequent to the homology clustering process (see below). A sub-group of 35 genomes was automatically annotated using the genome annotation system GenDB [42]. These annotations, together with the manually curated *A. salmonicida* genome, were used as templates to determine the function of genes from all 64 genomes. For homology clusters that contained at least one *A. salmonicida* gene product, the annotation from *A. salmonicida* was transferred to all sequences in the cluster. Homology clusters that did not contain a *A. salmonicida* reference gene were annotated based on the automatic annotations from GenDB and afterwards manually curated.

The number of different loci that unique core genes are distributed over was determined by choosing one isolate per unique core genome and investigate the location of unique core genes in it. To avoid over-estimation of genetic loci due to fragmented genome sequences we either chose a fully assembled genome sequence or, where just draft genome sequences where included in a unique core genome, the sequence with the lowest number of contigs per isolate.

### Homolog clustering and calculation of unique core genomes

Clustering of homologous protein sequences was performed using the freely available software orthoMCL. Although the orthoMCL algorithm shows a high degree of specificity and sensitivity, varying results can be achieved for the same datasets depending on the parameter values chosen [43].

In our analysis we chose a conservative value for the percent identity cut-off of 50% and set the E-value cut-off to *1e-05*. To minimize the effects of the remaining software parameters *percent match* and *inflation value*, we performed a total of 15 different orthoMCL runs with varying parameter values. The percent match parameter was set to 0,30,50 and 70 and the inflation value parameter was set to 0,3,5 and 7. A total of 12,914 homolog clusters containing genes of 2–63 isolates were conserved and stable over all 15 conditions including 74% (201,329) of all predicted protein coding sequences.

Determination of the different unique core genomes was based on the homology clusters found by orthoMCL. We assigned a number to the genome sequence of isolate in our dataset. The homology clusters that contained genes of the exact same combination of isolate numbers were then grouped together to the unique core genome of the particular combination of isolates. The size of the unique core genome is the number of homology cluster found for the combination of isolates.

It should be mentioned that the number of homologs, as well as the number of unique core genes, can be interpreted as a conservative lower boundary. This is due to the fact that the majority of genome sequences in our dataset are still draft genomes and the sequencing quality of some, especially *V. cholerae* genomes, was found to be very poor.

### Phylogenetic analysis

Genes for the multilocus sequence analysis were selected based on criteria widely accepted for phylogenetic inference [19,44-46]. We selected single copy genes present in all 64 genomes with a length of roughly 900–2500 nucleotides. Additionally only gene sequences were selected that were complete over the entire length in all genomes, i.e. genes from draft genomes with gaps or missing start/stop were also excluded from the alignments. Furthermore we excluded all genes of unknown function or annotated as hypothetical proteins to minimize the chance of chosing false positives. Based on these criteria we chose the nucleotide sequences of the six genes *uvrDdefBrsmBpmbAglnA* and *dapA*.

The concatenated sequences of all genes were aligned using MAFFT v. 6.833 [20] with default parameter (see Additional file 4). The maximum-Likelihood (ML) tree was generated using RaxML v. 7.0.4 and the GTR + G model [22]. The topology was next tested using 1,000 bootstrap replicates.

Bayesian analysis was performed using MrBayes v. 3.1.2 [23,24] with gamma distribution of rates, 5,000,000 generations and a sample frequency of 1,000. The burn in was set to 25%. The complete analysis was performed using the Epos framework v.0.9 [21].

## Competing interests

No competing interests for any of the authors exist.

## Author’s contributions

TK performed the computational analysis and interpretation of the data and drafted the manuscript. AG supervised the annotation of 35 sample genomes and contributed to the manuscript. EH contributed in data gathering, data analysis and the manuscript. NPW contributed to the design of the study and to the manuscript. PH supervised the study, contributed to the interpretation of the data and helped with drafting the manuscript. All authors read and approved the final manuscript.

## Supplementary Material

Additional file 1 **Genomes used in this study Office word document TableS1.doc.** Complete list of all bacterial strains used in the analysis. Click here for file

Additional file 2 **Unique core genomes of genophyletic groups of isolates. Office word document TableS2.doc.** Annotation summary of 46 unique core genomes of genophyletic groups that contain more than 10 genes per isolate. Unique core genomes that contain genes with functions related to pathogenicity are highlighted in grey. (1) Numbers in brackets denote the number of proteins involved in the given function. (2) Estimate of genetic loci the genes are distributed over in the chosen template isolate sequence. Click here for file

Additional file 3 **Detailed annotation summary of unique core genomes of genophyletic groups.** Office word document TableS3.doc. Table of unique core genomes of genophyletic groups of isolates containing the complete names of isolates included. Additionally, more detailed annotation remarks are shown. Click here for file
