# Supplementary material for: Unique core genomes of the bacterial family vibrionaceae: insights into niche adaptation and speciation
Source: BMC Genomics. 2012 May 10;13:179. doi: 10.1186/1471-2164-13-179 (PMC3464603; doi:10.1186/1471-2164-13-179)
Supplement: Additional file 1 — Genomes used in this study Office word document TableS1.doc. Complete list of all bacterial strains used in the analysis. [file 1471-2164-13-179-S1.doc]

| Organism | Genome size (MB) | GC content (%) | CDS | Draft | Number of contigs / replicons | Environmental isolate | Pathogenic | Genbank accession numbers |
| --- | --- | --- | --- | --- | --- | --- | --- | --- |
| *A. fischeri* str. ES114 | 4.28 | 38 | 3918 | No | 3 | Yes | No | CP000020  CP000021  CP000022 |
| *A. fischeri* str. MJ11 | 4.48 | 38 | 4028 | Yes | 38 | Yes | No | CP001133  CP001139  CP001134 |
| *A. salmonicida* str. LFI1238 | 4.65 | 38 | 4285 | No | 6 | Yes | Yes | FM178379  FM178380  FM178381  FM178382  FM178383  FM178384 |
| *A. wodanis* str. 06/09/139 | 4.48 | 39 | 4163 | Yes | 413 | Yes | Yes | NA |
| *V. alginolyticus* str. 12G01 | 5.16 | 44 | 4681 | Yes | 106 | Yes | Yes | AAPS00000000 |
| *V. alginolyticus* str. 40B | 5.14 | 44 | 4980 | Yes | 188 | Yes | No | ACZB00000000 |
| *V. anguillarum* str. NB10 | 4.17 | 44 | 3948 | Yes | 227 | Yes | Yes | NA |
| *V. campbellii* str. AND4 | 4.25 | 44 | 3891 | Yes | 143 | Yes | No | ABGR00000000 |
| *V. cholerae* str. 12129-1 | 3.96 | 47 | 3654 | Yes | 12 | Yes | Yes | ACFQ00000000 |
| *V. cholerae* str. 1587 | 4.13 | 47 | 3909 | Yes | 254 | Yes | Yes | AAUR00000000 |
| *V. cholerae* str. 2740-80 | 4.13 | 47 | 3698 | Yes | 254 | Yes | No | AAUT00000000 |
| *V. cholerae* str. 623-39 | 3.97 | 47 | 3790 | Yes | 314 | NA | Yes | AAWG00000000 |
| *V. cholerae* str. AM-19226 | 4.05 | 47 | 3716 | Yes | 154 | No | Yes | AATY00000000 |
| *V. cholerae* str. B33 | 4.02 | 47 | 3817 | Yes | 369 | NA | Yes | AAWE00000000 |
| *V. cholerae* str. biovar albensis VL426 | 3.98 | 47 | 3578 | Yes | 5 | Yes | Yes | ACHV00000000 |
| *V. cholerae* str. BX 330268 | 4 | 47 | 3643 | Yes | 8 | Yes | Yes | ACIA00000000 |
| *V. cholerae* str. CIRS 101 | 4.05 | 47 | 3724 | Yes | 18 | Yes | Yes | ACVW00000000 |
| *V. cholerae* str. CT5369-39 | 3.55 | 47 | 3487 | Yes | 269 | Yes | Yes | ADAL00000000 |
| *V. cholerae* str. INDRE 91-1 | 3.94 | 47 | 3633 | Yes | 60 | Yes | Yes | ADAK00000000 |
| *V. cholerae* str. M66-2 | 3.93 | 47 | 3538 | No | 2 | Yes | Yes | CP001233  CP001234 |
| *V. cholerae* str. MAK 757 | 3.91 | 47 | 3608 | Yes | 206 | No | Yes | AAUS00000000 |
| *V. cholerae* str. MJ1236 | 4.23 | 47 | 3852 | No | 2 | No | Yes | CP001485  CP001486 |
| *V. cholerae* str. MO10 | 4.03 | 47 | 3753 | Yes | 153 | No | Yes | AAKF00000000 |
| *V. cholerae* str. MZO-2 | 3.86 | 47 | 3507 | Yes | 162 | No | Yes | AAWF00000000 |
| *V. cholerae* str. MZO-3 | 4.14 | 47 | 3849 | Yes | 292 | No | Yes | AAUU00000000 |
| *V. cholerae* str. NCTC 8457 | 4.06 | 47 | 3974 | Yes | 390 | No | Yes | AAWD00000000 |
| *V. cholerae O1 biovar El Tor* str. N16961 | 4.03 | 47 | 3691 | No | 2 | No | Yes | AE003852  AE003853 |
| *V. cholerae* str. O395 | 4.13 | 47 | 3812 | No | 2 | No | Yes | CP000626  CP000627 |
| *V. cholerae* str. RC27 | 4.01 | 47 | 3730 | Yes | 45 | Yes | Yes | ADAI00000000 |
| *V. cholerae* str. RC385 | 3.64 | 47 | 3609 | Yes | 550 | Yes | Yes | AAKH00000000 |
| *V. cholerae* str. RC9 | 4.21 | 47 | 3890 | Yes | 11 | Yes | Yes | ACHX00000000 |
| *V. cholerae* str. TMA 21 | 4.02 | 47 | 3652 | Yes | 20 | Yes | No | ACHY00000000 |
| *V. cholerae* str. V51 | 3.78 | 47 | 3659 | Yes | 360 | No | Yes | AAKI00000000 |
| *V. cholerae* str. V52 | 3.97 | 47 | 3687 | Yes | 268 | No | Yes | AAKJ00000000 |
| *V. coralliilyticus* str. ATCC BAA-450 | 5.68 | 45 | 5217 | Yes | 20 | Yes | Yes | ACZN00000000 |
| *V. furnissi* str. CIP-102971 | 4.95 | 50 | 4569 | Yes | 24 | No | Yes | ACZP00000000 |
| *V. harveyi* str. 1DA3 | 5.93 | 45 | 5424 | Yes | 140 | Yes | No | ACZC00000000 |
| *V. harveyi* str. ATCC BAA-1116 | 6.05 | 45 | 5526 | No | 3 | Yes | Yes | CP000789  CP000790  CP000791 |
| *V. harveyi* str. HY01 | 5.4 | 45 | 5002 | Yes | 349 | Yes | Yes | AAWP00000000 |
| *V. metschnikovii* str. CIP 69-14 | 3.81 | 44 | 3473 | Yes | 11 | No | No | ACZO00000000 |
| *V. mimicus* str. VM223 | 4.34 | 46 | 4078 | Yes | 8 | Yes | Yes | ADAJ00000000 |
| *V. mimicus* str. VM573 | 4.36 | 46 | 4155 | Yes | 74 | No | Yes | ACYV00000000 |
| *V. mimicus* str. VM603 | 4.35 | 46 | 4036 | Yes | 195 | Yes | Yes | ACYU00000000 |
| *V. orientalis* str. CIP 102891 | 4.69 | 44 | 4256 | Yes | 5 | Yes | No | ACZV00000000 |
| *V. parahaemolyticus* str. 16 | 4.48 | 46 | 4179 | Yes | 178 | Yes | Yes | ACCV00000000 |
| *V. parahaemolyticus* str. AQ3810 | 5.77 | 45 | 6097 | Yes | 1073 | No | Yes | AAWQ00000000 |
| *V. parahaemolyticus* str. AQ4037 | 4.93 | 45 | 4573 | Yes | 167 | Yes | Yes | ACFN00000000 |
| *V. parahaemolyticus* str. K5030 | 5.02 | 45 | 4677 | Yes | 164 | NA | Yes | ACKB00000000 |
| *V. parahaemolyticus* str. Peru-466 | 5.03 | 45 | 4640 | Yes | 149 | NA | Yes | ACFM00000000 |
| *V. parahaemolyticus* str. RIMD 2210633 | 5.16 | 45 | 4686 | No | 2 | No | Yes | BA000031  BA000032 |
| *V. shilonii* str. AK1 | 5.7 | 43 | 5315 | Yes | 158 | Yes | Yes | ABCH00000000 |
| *V. sp.* EX25 | 4.84 | 44 | 4451 | Yes | 222 | Yes | Yes | AAKK00000000 |
| *V. splendidus* str. 12B01 | 5.59 | 44 | 4962 | Yes | 119 | Yes | Yes | AAMR00000000 |
| *V. splendidus* str. LGP32 | 4.97 | 44 | 4361 | No | 2 | Yes | Yes | FM954972  FM954973 |
| *V. sp.* MED222 | 4.89 | 43 | 4290 | Yes | 99 | Yes | No | AAND00000000 |
| *V. sp.* RC341 | 4.00 | 46 | 3690 | Yes | 28 | Yes | No | ACZT00000000 |
| *V. sp.* RC586 | 4.08 | 46 | 3713 | Yes | 16 | Yes | No | ADBD00000000 |
| *V. vulnificus* str. YJ016 | 5.26 | 46 | 4755 | No | 3 | No | Yes | BA000037  BA000038  AP005352 |
| *V. vulnificus* str. CMCP6 | 5.12 | 46 | 4767 | No | 2 | No | Yes | AE016795  AE016796 |
| *P. angustum* str. S14 | 5.1 | 39 | 4579 | Yes | 45 | Yes | No | AAOJ00000000 |
| *P. damselae* str. CIP 102761 | 5.04 | 41 | 4500 | Yes | 8 | Yes | Yes | ADBS00000000 |
| *P. profundum* str. 3tck | 6.1 | 41 | 5553 | Yes | 82 | Yes | No | AAPH00000000 |
| *P. profundum* str. S99 | 6.4 | 41 | 5952 | No | 3 | Yes | No | CR354531  CR354532  CR377818 |
| *P. sp.* SKA34 | 4.94 | 39 | 4579 | Yes | 88 | Yes | No | AAOU00000000 |
