# Supplementary material for: Unique core genomes of the bacterial family vibrionaceae: insights into niche adaptation and speciation
Source: BMC Genomics. 2012 May 10;13:179. doi: 10.1186/1471-2164-13-179 (PMC3464603; doi:10.1186/1471-2164-13-179)
Supplement: Additional file 2 — Unique core genomes of genophyletic groups of isolates. Office word document TableS2.doc. Annotation summary of 46 unique core genomes of genophyletic groups that contain more than 10 genes per isolate. Unique core genomes that contain genes with functions related to pathogenicity are highlighted in grey. (1) Numbers in brackets denote the number of proteins involved in the given function. (2) Estimate of genetic loci the genes are distributed over in the chosen template isolate sequence. [file 1471-2164-13-179-S2.doc]

| **# of isolates included** | **# of homolog clusters** | **Phage/plasmid related proteins** | **Hypothetical proteins** | **Loci of different known function^1^** | **# of genetic loci^2^** |
| --- | --- | --- | --- | --- | --- |
| 2 | 12 | - | 4 | - | >5 |
| 2 | 12 | - | 3 | incf plasmid conjugative transfer proteins (7) | 1 |
| 2 | 12 | - | 10 |  | 2 |
| 2 | 15 | - | 9 |  | >5 |
| 2 | 15 | - | 13 | - | 3 |
| 2 | 31 |  | - | PTS system  Sugar metabolism | >5 |
| 2 | 28 | 10 |  | - | 2 |
| 2 | 29 | 5 | 20 | - | >5 |
| 2 | 28 | - | 9 | - | 5 |
| 1 | 25 | 10 | 15 | - | 1-2 |
| 2 | 24 | - | 11 | - | >5 |
| 2 | 23 | - | 3 | - | >5 |
| 2 | 22 | - | 14 | - | <=5 |
| 2 | 22 | - | - | Flagellar genes (15) | >5 |
| 2 | 17 |  | 10 | - | >=5 |
| 2 | 17 | - | 8 | Fimbria/pilus related (3) | >5 |
| 2 | 16 | - | 13 | - | 1 |
| 2 | 16 | - | 10 | - | >5 |
| 2 | 15 | - | 6 | - | <=5 |
| 2 | 14 | - | 10 | - | 4 |
| 2 | 14 | - | 2 | - | >5 |
| 2 | 13 | 6 | 7 | - | 2 |
| 2 | 13 | - | 11 | - | >5 |
| 2 | 92 | 92 | - | - | 1 |
| 3 | 14 | 9 | 5 | - | 3 |
| 3 | 13 | - | 7 | - | >5 |
| 3 | 12 | - | 10 | - | 2 |
| 3 | 12 | - | 10 | - | 2 |
| 3 | 11 | - | 3 | - | 1 |
| 3 | 11 | - | 3 | - | >5 |
| 3 | 12 | - | 6 | - | 3 |
| 4 | 12 | - | 2 | Type VI secretion system (10) | 2 |
| 4 | 11 | - | 3 | - | >=5 |
| 4 | 17 | 14 | 3 | - | 1 |
| 5 | 19 | - | - | Hydrogenase/formate dehydrogenase biosynthesis (19) | 1 |
| 6 | 15 | - | 1 | Uber-operon | 1 |
| 6 | 12 | - | 3 | - | 1 |
| 6 | 13 | - | 3 | Purine metabolism | 1 |
| 11 | 11 | - | 4 | TTSS (5) | 1 |
| 12 | 15 | - | 1 | ABC-transporter (5) | 2 |
| 20 | 12 | - | - | Flagellar related | 1 |
| 13 | 27 | - | - | TTSS | 1 |
| 14 | 14 | - | 7 | Type IV secretion system | 1 |
| 17 | 15 | - | 1 | Tcp gene cluster | 1 |
| 19 | 17 | - | - | Lateral flagellar system | 2 |
| 62 | 15 | - | 1 | - | <=5 |
