# Supplementary material for: Unique core genomes of the bacterial family vibrionaceae: insights into niche adaptation and speciation
Source: BMC Genomics. 2012 May 10;13:179. doi: 10.1186/1471-2164-13-179 (PMC3464603; doi:10.1186/1471-2164-13-179)
Supplement: Additional file 3 — Detailed annotation summary of unique core genomes of genophyletic groups. Office word document TableS3.doc. Table of unique core genomes of genophyletic groups of isolates containing the complete names of isolates included. Additionally, more detailed annotation remarks are shown. [file 1471-2164-13-179-S3.doc]

| **# of isolates** | **# of homolog clusters** | **Name of isolates included in the unique core genome** | **Annotation remarks** |
| --- | --- | --- | --- |
| 2 | 92 | *V. cholerae str. RC9*  *V. mimicus str. VM603* | One locus/region with few genes missing in between  Most likely plasmid, no attC sites found  Contains 59 hypothetical proteins  Containing genes:  - Exonuclease (Ycg4D)  - DNA replication terminus site-binding protein  - KfrA protein  - putative DNA helicase  - type IV conjugative transfer system protein TraF  - type IV conjugative transfer system protein TraG  - DNA topoisomerase III  - DNA modification methylase  - cytosine-specific DNA methylase  - RHS repeat-associated core domain protein |
| 2 | 31 | *P. profundum str. 3tck*  *V. sp. str. EX25* | Genes distributed over multiple loci/regions  5 hypothetical proteins  10 involved in sugar metabolism or part of a phosphotransferase system (PTS) including:   - PTS system, fructose(mannose)-specific IIB, IIC and IICD - PTS system, IIA component, putative - maltose regulon regulatory protein MalI - mannose-6-phosphate isomerase-like protein - PTS system fructose-like IIB component 1 - PTS system fructose-like IIC - glucose-6-phosphate isomerase |
| 2 | 28 | *V. Anguillarum str. NB10*  *V. cholerae str. V51* | Most genes found in the same locus/region  10 phage or prophage -related proteins including   - prophage LambdaSo, minor tail protein M - phage minor tail protein L - lambda tail assembly I - Phage-related minor tail protein - prophage LambdaSo, major tail protein V - possible phage head-tail adaptor - phage portal protein, HK97 family - phage terminase, large subunit, putative |
| 2 | 29 | *V. harveyi str. ATCC BAA-1116*  *V. campbellii str. AND4* | Genes distributed over multiple loci/regions  20 hypothetical proteins  5 phage related proteins including:   - phage protein U-like protein - phage protein D-like protein - phage-related tail protein - phage tail sheath protein FI-like protein - putative tail tube protein |
| 2 | 28 | *V. orientalis str. CIP102891*  *V. shilonii str. AK1* | Genes distributed over multiple loci/regions  9 hypothetical proteins  No phage –related proteins  Genes distributed over several regions/loci  Two possible operons containing 10 and 12 genes  Region 1 (10 genes)   - glutamine synthetase family protein - glutamine synthetase - putrescine aminotransferase - putrescine transporter subunit - aldehyde dehydrogenase   Region 2 (12 genes)   - type 1 pili chaperone protein FimC - putative outer membrane protein - Fimbrial proteins - Sensory box sensor histidine kinase/response regulator VieS - Response regulator VieA - putative fimbrial protein Z, transcriptional regulator |
| 13 | 27 | *V. alginolyticus str. 12G01*  *V. alginolyticus str. 40B*  *V. harveyi str. 1DA3*  *V. harveyi str. ATCC BAA-1116*  *V. harveyi str. HY01*  *V. parahaemolyticus str. AQ3810*  *V. parahaemolyticus str. AQ4037*  *V. parahaemolyticus str. K5030*  *V. parahaemolyticus str. Peru-466*  *V. parahaemolyticus str. RIMD 2210633*  *V. campbellii str. AND4*  *V. sp. EX25*  *P. damselae str. CIP 102761* | Genes located in one region/locus  Most genes related to TTSS including:   - type III secretion thermoregulatory protein - type III secretion system chaperone - type III secretion outer membrane pore - type III secretion apparatus protein - type III secretion apparatus needle protein - type III secretion protein - putative type III export protein - type III export protein - type III secretion apparatus lipoprotein - type III secretion cytoplasmic protein - type III secretion inner membrane protein - type III secretion low calcium response chaperone - translocator protein PopB - translocator protein PopD |
| 2 | 25 | *V. cholerae str. NCTC-8457*  *V. sp RC586* | All but last 5 in the same region (maybe same region on 2 contigs?)  15 hypothetical proteins  10 phage-related proteins including:   - prophage MuMc02, tail sheath protein - phage proteins - putative phage baseplate protein - phage tail protein I - tail fiber protein H, putative - phage tail tape measure protein TP901 family - baseplate assembly protein J - probable bacteriophage regulatory protein, putative |
| 2 | 24 | *P. angustum str. S14*  *P. damselae str. CIP-102761* | Genes distributed over multiple loci/regions  11 hypothetical proteins  No phage –related proteins  Proteins of various unrelated functions including:   - formate dehydrogenase accessory protein - formate dehydrogenase N, nitrate-inducible, cytochrome B556(Fdn) gamma subunit - outer membrane protein OmpU - is606 transposase - iron-containing alcohol dehydrogenase |
| 2 | 23 | *V. furnissi str. CIP-102971*  *V. metschnikovii_str. CIP 69-14* | Genes distributed over multiple loci/regions  3 hypothetical proteins  No phage –related proteins  Proteins of various unrelated functions including:   - probable transcription regulators - arginine N-succinyltransferase - na+-driven multidrug efflux pump - DNA polymerase III epsilon subunit - lysine exporter protein - methyl-accepting chemotaxis sensory transducer - alanine racemase biosynthetic - ABC transporter ATP-binding proteins |
| 2 | 22 | *V. cholerae str. CT 5369-93*  *V. mimicus str. VM223* | Genes distributed over multiple loci/regions  14 hypothetical proteins  No phage –related proteins  Proteins of various unrelated functions including:   - glycosyl transferase group 1 - coenzyme F420-dependent oxidoreductase   3 proteins related to secretion system including:   - bacterial type II and III secretion system family protein - general secretion pathway protein F - S-protein secretion component E |
| 2 | 22 | *V. furnissi str. CIP-102971*  *V. shilonii str. AK1* | Genes distributed over multiple loci/regions  No hypothetical proteins  No phage-related proteins  8 genes of various unrelated function including:   - L-rhamnose mutarotase - transcriptional regulator - uracil-xanthine permease - CBS domain containing membrane protein   One region containing 15 flagellar-related genes including:   - FliF, FliG, FliP, FliR and FliS - FlgH and FlgI - FlgB, FlgC, FlgD, FlgF, FlgG - FlhB |
| 5 | 19 | *V. furnissi str. CIP-102971*  *P. angustum str. S14*  *P. damselae str. CIP-102761*  *P. profundum str. 3tck*  *P. sp. SKA34* | All but one gene located in one locus/region  No hypothetical proteins  No phage-related proteins  All genes involved in hydrogenase and formate hydroganelyase biosynthesis including:   - hydrogenase nickel incorporation-associated protein HypB, HypD, HypE and HypF - hydrogenase nickel incorporation protein HybF - formate dehydrogenase chain D - formate hydrogenlyase transcriptional activator - formate dehydrogenase H - hydrogenase 3 maturation protease - formate hydrogenlyase maturation protein hycH - formate hydrogenlyase subunit 7 - formate hydrogenlyase complex 3 iron-sulfur protein - hydrogenase 4 subunit - hydrogenase-4 component C |
| 4 | 17 | *V. campbellii str. AND4*  *V. splendidus str. 12G01*  *P. damselae str. CIP 102761*  *A. wodanis str. 06-09-139* | Genes located in the same region/locus  3 hypothetical proteins  Most proteins phage/prophage related function including:   - phage terminase large subunit - putative portal protein - prophage LambdaW5, minor tail protein Z, putative - Phage baseplate assembly protein V - Baseplate J-like protein - Phage tail protein I - Phage tail sheath protein FI-like - putative tail tube protein - putative phage-related protein - tail tape measure protein - Phage protein U-like - phage tail X - Phage protein D-like |
| 2 | 17 | 1. *fischeri str. MJ11*   *A. wodanis str. 06-09-139* | Genes distributed over multiple loci/regions  8 hypothetical proteins  No phage-related proteins  Proteins of various unrelated functions including:   - filamentation induced by cAMP protein Fic - plasmid maintenance system killer protein - addiction module antidote protein, HigA family - fimbrial protein pilin - type 4 fimbrial biogenesis protein FimT - type IV pilus assembly protein PilW |
| 2 | 17 | *V. coralliilyticus str. ATCC BAA-450*  *V. splendidus str. 12G01* | Genes distributed over multiple loci/regions  10 hypothetical proteins  No phage-related proteins  Proteins of various unrelated functions including:   - putative Secretion protein - AcrB/AcrD/AcrF family protein - probable two-component sensor - methylation site containing protein - putative sensor histidine kinase |
| 19 | 17 | *V. alginolyticus str. 12G01*  *V. alginolyticus str. 40B*  *V. coralliilyticus ATCC BAA-450*  *V. furnissi str. CIP-102971*  *V. harveyi str. 1DA3*  *V. harveyi str. ATCC BAA-1116*  *V. harveyi str. HY01*  *V. mimicus str. VM573*  *V. mimicus str. VM 603*  *V. parahaemolyticus str. AQ3810*  *V. parahaemolyticus str. AQ4037*  *V. parahaemolyticus str. K5030*  *V. parahaemolyticus str. Peru-466*  *V. parahaemolyticus str. RIMD 2210633*  *V. campbellii str. AND4*  *V. shilonii str. AK1*  *V. sp. EX25*  *P. angustum str. S14*  *P. sp. SKA34* | Genes located in the same region/locus  No hypothetical proteins  No phage-related proteins  Genes from lateral flagellar system of *V. parahaemolyticus* including:   - LafU, LafT,LafS, LafF, LafC, LafA - Lateral flagellar hook-associated protein 2 - flagellar biosynthetic protein - flagellar M-ring protein - flagellar motor switch protein G - flagellar assembly protein FliH - flagellar hook-associated protein L - putative flagellar hook-associated protein - putative flagellar basal-body rod protein - Flagellar hook protein FlgE - flagellar basal-body rod modification protein D |
| 2 | 16 | *V. alginolyticus str. 12G01*  *V. anguillarum str. NB10* | Most genes located in the same region/locus  13 hypothetical proteins |
| 2 | 16 | *V. harveyi str. HY01*  *V. campbellii str. AND4* | Genes distributed over multiple loci/regions  10 hypothetical proteins  No phage-related proteins  Proteins of various unrelated functions including:   - putative lipoproteins - protein SopB - plasmid partitioning transcription repressor - putative Mcf2 |
| 62 | 15 | All but  *V. cholerae str. RC385*  *V. cholerae str. V51* | Genes distributed over multiple loci/regions  1 hypothetical protein  No phage-related proteins  2 regions of 5 genes each with genes next/close to each other  Region 1 including:   - dihydrofolate reductase type 3 - diadenosine tetraphosphatase - dimethyladenosine transferase - 4-hydroxythreonine-4-phosphate dehydrogenase - chaperone SurA precursor   Region 2 including:   - putative membrane proteins - methyltransferase(s) - cytochrome D ubiquinol oxidase subunit I |
| 2 | 15 | *V. harveyi str. 1DA3*  *V. shilonii str. AK1* | Genes distributed over multiple loci/regions  9 hypothetical protein  No phage-related proteins  Proteins of various unrelated functions including:   - sucrose-6-phosphate hydrolase - DNA-binding transcriptional repressor RpiR - beta-lactamase-like protein - MarR family transcriptional regulator - transcriptional regulator, LysR family protein |
| 2 | 15 | *V. mimicus str. VM223*  *V. sp. RC341* | Genes located in the same region/locus  13 hypothetical proteins  No phage-related functions |
| 6 | 15 | *V. cholerae str. RC385*  *V. mimicus str. VM223*  *V. mimicus str. VM573*  *V. mimicus str. VM603*  *V. shilonii str. AK1*  *V. vulnificus str. YJ016* | Genes located in the same region/locus  1 hypothetical protein  No phage-related proteins  Proteins of various functions might include *V. vulnificus*  uber-operon 74 from Uber-operon database (<http://csbl.bmb.uga.edu/uber/index.php>):   - operon 263 - operon 264 - operon 265 |
| 17 | 15 | *V. cholerae str. B33*  *V. cholerae str. BX 330286*  *V. cholerae str. CIRS 101*  *V. cholerae str. INDRE 91-1*  *V. cholerae str. M66-2*  *V. cholerae str.MAK757*  *V. cholerae str. MJ1236*  *V. cholerae str. MO10*  *V. cholerae str. NCTC 8457*  *V. cholerae str. O! biovar eltor N16961*  *V. cholerae str. O395*  *V. cholerae str. RC27*  *V. cholerae str. RC9*  *V. cholerae str. V51*  *V. cholerae str. V52*  *V. cholerae str. 2740-80*  *V. mimicus VM573* | Genes located in the same region/locus  1 hypothetical protein  No phage-related proteins  Region including the complete toxin co-regulated pilus (tcp) gene cluster as described in P.A. *Manning, 1997, ‘The tcp gene cluster of V. cholerae’*  except genes TcpA and TcpJ |
| 2 | 15 | *V. coralliilyticus str. ATCC BAA-450*  *V. parahaemolyticus str. 16* | Genes distributed over multiple loci/regions  6 hypothetical protein  No phage-related proteins  Proteins of various unrelated functions including:   - transcriptional regulator(s) - extracellular solute-binding protein, family 3 - acetyltransferase including N-acetylase of ribosomal protein - autoinducer 1 sensor kinase/phosphatase luxN - sensory box (GGDEF/EAL domain) regulatory protein |
| 12 | 15 | *V. alginolyticus str. 12G01*  *V. alginolyticus str. 40B*  *V. coralliilyticus str. ATCC BAA-450*  *V. harveyi str. 1DA3*  *V. orientalis str. CIP 102891*  *V. parahaemolyticus str. 16*  *V. parahaemolyticus str. AQ3810*  *V. parahaemolyticus str. AQ4037*  *V. parahaemolyticus str. K5030*  *V. parahaemolyticus str. Peru-466*  *V. parahaemolyticus str. RIMD 2210633*  *V. sp. EX25* | Genes distributed over 2 loci/regions  1 hypothetical protein  No phage-related proteins  Region 1 including:   - 5 oligopeptide ABC transporter   Region 2 including proteins of various functions:   - putative aminopeptidase - collagenase - probable dihydrodipicolinate synthetase - ornithine cyclodeaminase - probable binding protein component of ABC transporter - proline racemase - fatty aldehyde dehydrogenase - putative transcription regulator |
| 14 | 14 | *V. alginolyticus str. 12G01*  *V. alginolyticus str. 40B*  *V. coralliilyticus str. ATCC BAA-450*  *V. harveyi str. 1DA3*  *V. harveyi str. ATCC BAA-1116*  *V. harveyi str. HY01*  *V. parahaemolyticus str. AQ4037*  *V. parahaemolyticus str. K5030*  *V. parahaemolyticus str. Peru-466*  *V. parahaemolyticus str. RIMD 2210633*  *V. campbellii str. AND4*  *V. sp. EX25*  *V. splendidus str. LPG32*  *A. fischeri str. MJ11* | Genes located in the same region/locus  7 hypothetical protein  No phage-related proteins  Most genes related to type VI secretion system including:   - type VI secretion protein - type VI secretion system IcmF - type VI secretion system lysozyme-like protein - type VI secretion system-associated - putative transcriptional regulator(s) |
| 3 | 14 | *V. harveyi str. ATCC BAA-1116*  *V. campbellii str. AND4*  *V. splendidus str. 12B01* | Genes located in the same region/locus  5 hypothetical protein  9 phage/prophage related protein:   - prophage PSPPH06, putative tail tube protein - prophage PSPPH06, putative tail sheath protein - prophage PSPPH06, virion morphogenesis protein - prophage PSPPH06, putative head completion/stabilization protein |
| 2 | 14 | *V. cholerae str. B33*  *V. cholerae str. MJ1236* | Genes located in the same region/locus  10 hypothetical protein  No phage-related proteins  Proteins of various unrelated functions including:   - tetracycline efflux protein - glyoxalase/bleomycin resistance protein/dioxygenase - truncated helix-turn-helix multiple antibiotic resistance protein |
| 2 | 14 | *V. furnissi str. CIP-102971*  *V. parahaemolyticus str. 16* | Genes distributed over multiple loci/regions  2 hypothetical protein  No phage-related proteins  Proteins of various unrelated functions including:   - TRAP dicarboxylate transporter DctM subunit - universal stress protein - lysine exporter protein (LYSE/YGGA) - putative glycosyltransferase - capsular polysaccharide export system protein KpsS - transcriptional regulator LysR family - nitrilotriacetate monooxygenase component A |
| 2 | 13 | *V. alginolyticus str. 12G01*  *V. harveyo str. HY01* | Genes located in the same region/locus  7 hypothetical protein  6 phage-related proteins including:   - minor tail protein - possible bacteriophage tail protein - Putative tail fiber component V of prophage - hypothetical protein (probably prophage associated) - minor tail protein T - tail assembly protein, putative |
| 6 | 13 | *V. coralliilyticus str. ATCC BAA-450*  *V. splendidus str. 12B01*  *V. shilonii str. AK1*  *V. orientalis str. CIP 102891*  *P. profundum str. 3tck*  *P. profundum S99* | Genes located in the same region/locus  3 hypothetical proteins  No phage-related proteins  Several proteins involved in purine-matabolism including:   - xanthine dehydrogenase, XdhA subunit - xanthine dehydrogenase accessory factor XdhC - ureidoglycolate hydrolase - xanthine/uracil permease - putative glyoxylate carboligase |
| 3 | 13 | *V. harveyi str. ATCC BAA-1116*  *V. harveyi str. HY01*  *V. campbellii str. AND4* | Genes distributed over multiple loci/regions  7 hypothetical protein  No phage-related proteins  Proteins of various unrelated functions including:   - sensory transduction protein kinase - transcriptional regulator(s) - protease |
| 2 | 13 | *V. cholerae str. RC385*  *V. sp str. RC586* | Genes distributed over multiple loci/regions  11 hypothetical protein  No phage-related proteins |
| 3 | 12 | *V. shilonii str. AK1*  *A. salmonicida str. LFI1238*  *P. profundum str. 3tck* | Genes distributed over multiple loci/regions  12 hypothetical protein  No phage-related proteins |
| 3 | 12 | *V. cholerae str. 1587*  *V. cholerae str. 623-39*  *V. parahaemolyticus str. AQ4037* | Genes distributed over multiple loci/regions  12 hypothetical protein  No phage-related proteins |
| 3 | 12 | *V. alginolyticus str. 12G01*  *V. alginolyticus str. 40B*  *V. harveyi str. 1DA3* | Genes distributed over multiple loci/regions  6 hypothetical protein  No phage-related proteins  Proteins of various unrelated functions including:   - Aspartate:alanine antiporter - aspartate aminotransferase - putative lipoprotein component of a efflux transporter |
| 2 | 12 | *V. coralliilyticus str. ATCC BAA-450*  *P. profundum str. 3tck* | Genes distributed over multiple loci/regions  4 hypothetical protein  No phage-related proteins  Proteins of various unrelated functions including:   - transcriptional regulator - ABC transporter permease component - glycerol-3-phosphate-binding periplasmic protein precursor - Phosphoglycolate phosphatase - putative Glutamate synthase GltB |
| 2 | 12 | *A. wodanis str. 06-09-139*  *P. damselae str. CIP 102761* | Genes located in the same region/locus  3 hypothetical protein  No phage-related proteins  Several genes related to plasmid transfer including:   - incf plasmid conjugative transfer pilus assembly protein traH, traN, traB and traE - incf plasmid conjugative transfer protein trbB and trb |
| 6 | 12 | *V. mimicus VM223*  *V. mimicus VM573*  *V. mimicus VM603*  *V. sp RC341*  *V. vulnificus str. CMCP6*  *V. vulnificus str. YJ016* | Genes located in the same region/locus  3 hypothetical protein  No phage-related proteins  Proteins of various functions including transport:   - ABC transporter ATPase - dipeptide/oligopeptide/nickel ABC transporter permease - oligopeptide ABC transporter permease - dipeptide ABC transporter periplasmic protein - arylsulfatase - N-acetylglucosamine-regulated outer membrane porin |
| 20 | 12 | *V. alginolyticus str. 12G01*  *V. alginolyticus str. 40B*  *V. coralliilyticus str. ATCC BAA-450*  *V. furnissi str. CIP-102971*  *V. harveyi str. 1DA3*  *V. harveyi str. ATCC BAA-1116*  *V. harveyi str. HY01*  *V. mimicus VM573*  *V. mimicus VM603*  *V. parahaemolyticus str. AQ3810*  *V. parahaemolyticus str. AQ4037*  *V. parahaemolyticus str. K5030*  *V. parahaemolyticus str. Peru-466*  *V. parahaemolyticus str. RIMD 2210633*  *V. campbellii str. AND4*  *V. sp. EX25*  *V. shilonii str. AK1*  *P. angustum str. S14*  *P. Profundum str. S99*  *P. sp SKA34* | Genes located in the same region/locus  No hypothetical protein  No phage-related proteins  All proteins flagellar-related function including:   - putative flagellar biosynthetic protein FliR - flagellar biosynthetic protein - putative flagellar assembly protein - flagellar motor switch protein - putative flagellar hook-basal body complex protein - flagellar P-ring protein precursor I and H - putative flagellar basal-body rod protein - flagellar basal-body rod protein FlgB and FlgC |
| 2 | 12 | *V. mimicus str. VM223*  *V. sp RC586* | Genes distributed over multiple loci/regions  9 hypothetical protein  No phage-related proteins  Proteins of various unrelated functions including:   - alanyl-tRNA synthetase domain protein - threonine efflux protein - biotin carboxylase |
| 4 | 12 | *V. anguilarum str. NB10*  *V. splendidus str. 12B01*  *A. salmonicida str. LFI1238*  *A. wodanis str. 06-09-139* | Genes located in the same region/locus  2 hypothetical protein  No phage-related proteins  10 genes part of type VI secretion system including:   - type VI secretion proteins VasA-1,VasB-1,VasC-1,VasD-1,VasE-1,VasF-1,VasV-1,VasS-1,VasJ-1,VasRB-1 |
| 4 | 11 | *V. splendidus str. 12B01*  *V. splendidus str. LGP32*  *V. shilonii str. AK1*  *V. sp MED222* | Genes distributed over multiple loci/regions  3 hypothetical protein  No phage-related proteins  Proteins of various mostly unrelated functions including:   - malate synthase G - isocitrate lyase - Glyoxalase/bleomycin resistance protein/dioxygenase - DNA-binding response regulator ColR, putative - sensor histidine kinase |
| 3 | 11 | *V. coralliluyticus str. ATCC BAA-450*  *V. orientalis str. CIP 102891*  *V. splendidus str. 12B01* | Genes located in the same region/locus  3 hypothetical protein  No phage-related proteins  Proteins of various mostly functions including:   - TPR repeat protein - cytochrome c biogenesis (thioredoxin) related protein - TadB-like protein involved in pilus formation and/or protein secretion - probable CpaE2 pilus assembly protein - type II and III secretion system protein - ApbE-like lipoprotein |
| 3 | 11 | *P. angustum str. S14*  *P. profundum str. 3tck*  *P. sp. SKA34* | Genes distributed over multiple loci/regions  3 hypothetical protein  No phage-related proteins  Proteins of various mostly unrelated functions including:   - methyl-accepting chemotaxis protein - Di-haem cytochrome c peroxidase - Rhodanese-like protein - MutT/nudix family protein |
| 11 | 11 | *V. cholerae str. AM-19226*  *V. cholerae str. TMA-21*  *V. cholerae str. V51*  *V. cholerae str. 1587*  *V. cholerae str. 623-39*  *V. cholerae str. 12129-1*  *V. parahaemolyticus str. AQ4037*  *V. parahaemolyticus str. AQ3810*  *V. parahaemolyticus str. K5030*  *V. parahaemolyticus str. Peru-466*  *V. parahaemolyticus str. RIMD 2210633* | Genes located in the same region/locus  4 hypothetical protein  No phage-related proteins  5 proteins related to TTSS including:   - Type III secretion protein Spa33 - type III secretion system EscU protein - type III secretion system EscV protein - type III secretion host injection protein (YopB) - type III secretion system lipoprotein EprK |
